# Once episiotomy, always episiotomy?

**DOI:** 10.1007/s00404-018-4783-8

**Published:** 2018-05-21

**Authors:** Ayala Zilberman, Eyal Sheiner, Orit Barrett, Batel Hamou, Tali Silberstein

**Affiliations:** 1 Soroka University Medical Center, Ben-Gurion University of the Negev, Beersheba, Israel; 2 Department of Obstetrics and Gynecology, Soroka University Medical Center, Ben-Gurion University of the Negev, Beersheba, Israel; 3 Department of Medicine and Clinical Research Center, Soroka University Medical Center, Ben-Gurion University of the Negev, Beersheba, Israel

**Keywords:** Episiotomy, Perineal tears, Perineal damage

## Abstract

**Objective:**

To investigate the association between episiotomy and perineal damage in the subsequent delivery.

**Study design:**

A retrospective cohort study was conducted, comparing outcome of subsequent singleton deliveries of women with and without episiotomy in their first (index) delivery. Deliveries occurred between the years 1991–2015 in a tertiary medical center. Traumatic vaginal tears, multiple pregnancies, and cesarean deliveries (CD) in the index pregnancy were excluded from the analysis. Multiple logistic regression models were used to control for confounders.

**Results:**

During the study period, 43,066 women met the inclusion criteria; of them, 50.4% (*n* = 21,711) had subsequent delivery after episiotomy and 49.6% (*n* = 21,355) had subsequent delivery without episiotomy in the index pregnancy. Patients with episiotomy in the index birth higher rates of subsequent episiotomy (17.5 vs. 3.1%; *P* < 0.001; OR 1.9; 95% CI). In addition, the rates of the first and second degree perineal tears as well as the third and fourth degree perineal tears were significantly higher in patients following episiotomy (33.6 vs. 17.8%; *P* < 0.001, and 0.2 vs. 0.1%; *P* = 0.002, respectively). Nevertheless, there was no significant difference at the rates of CD and instrumental deliveries, between the groups. While adjusting for maternal age, ethnicity, birth weight, and vacuum delivery—the previous episiotomy was noted as an independent risk factor for recurrent episiotomy in the subsequent delivery (adjusted OR 6.7; 95% CI 6.2–7.3, *P* < 0.001). The results remained significant for term (adjusted OR 6.8; 95% CI 6.2–7.4, *P* < 0.001) as well as preterm deliveries (adjusted OR 4.5; 95% CI 3.3–6.3, *P* < 0.001) in two different models.

**Conclusion:**

Episiotomy is an independent risk factor for recurrent episiotomy in the subsequent delivery.

## Introduction

The morbidity associated with perineal injury related to childbirth is a major health problem [1, 2]. Most vaginal births are associated with some form of trauma to the genital tract, either perineal tear or episiotomy, following spontaneous vaginal delivery [3]. Episiotomy is a common obstetric procedure, performed with scissors or scalpel, and is typically midline (median) or mediolateral in location [4]. It is considered when the clinical circumstances place the patient at high risk of a third or fourth degree laceration or when the fetal heart tracing is of concern and hastening vaginal delivery is warranted. Episiotomy may result in extension of the episiotomy incision and deformed anatomic outcomes, increased blood loss and hematoma formation, discomfort and pain, inflammation, infection and dehiscence within the episiotomy region, sexual dysfunction, and increased costs. It is unclear whether routine episiotomy improves the long-term risks of pelvic floor relaxation, pelvic organ prolapse, urinary incontinence, and dyspareunia [5, 6]. Moreover, mediolateral episiotomy found to be an independent risk factor for the third or fourth degree perineal tears even in critical conditions such as shoulder dystocia, instrumental deliveries, occiput-posterior position, fetal macrosomia, and non-reassuring fetal heart rate [1, 7]. Obstetricians’ perception that episiotomy decreases the risk of perineal trauma as compared with spontaneous tears constitutes the most substantial justification for this practice [8]. Restrictive use of episiotomy has been advocated given the risks of the procedure and unclear benefits of routine use [9]. In 2006, the American Congress of Obstetricians and Gynecologists recommended against routine episiotomy, and in 2008, the National Quality Forum recognized limiting routine episiotomy as an important measure of quality and patient safety, noting increased risks of pain, laceration, and anal incontinence with the procedure [10]. Since 2006, the episiotomy rate in the United States dropped from 17.3 to 11.6% in 2012 almost reaching 10% episiotomy rate that was recommended by the World Health Organization [11].

The aim of this study was to investigate the association between episiotomy in the first delivery and repeated episiotomy and perineal damage in the subsequent delivery.

## Methods

A retrospective cohort study was conducted, comparing outcomes of subsequent singleton deliveries of women with and without episiotomy in their first (index) delivery. Deliveries occurred at the Soroka University Medical Center between the years 1991 and 2015 were reviewed. Soroka University Medical Center is a tertiary medical center that serves the southern part of Israel. Data were retrieved from the perinatal computerized database. Traumatic vaginal tears, multiple pregnancies, and cesarean deliveries (CD) in the index pregnancy were excluded from the analysis.

Demographic and clinical characteristics were collected including maternal age, ethnicity (Jewish or Muslim), parity, smoking, diabetes mellitus, and hypertension. Obstetrical risk factors that were evaluated include: polyhydramnios, oligohydramnios, gestational diabetes mellitus (GDM), and premature rupture of membranes (PROM).

Birth characteristics and outcomes and delivery complications were assessed: spontaneous delivery, vacuum extraction, cesarean section, premature delivery, perineal tears, and episiotomy.

The following newborn characteristics were collected: gender, gestational age, and birth weight.

### Statistical analysis

Sociodemographic characteristics and comorbidities are presented as mean ± SD for normal distributed continuous variables or median with maximal and minimal values for non-normally distributed continuous variables. Categorical variables are presented as percentage. Categorical variables were compared using a Chi-square test. Continuous variables were examined using *t* test for normally distributed variables and by Mann–Whitney for non-parametric variables. Multiple logistic regression models were used to control for potential confounders. Variables that had a statistically significant in the univariate analysis, as well as variables having clinically significance, were included to the final multivariate regression. Quality of the final model was determined by − 2 log likelihood. An odds ratio (OR), *P* value, and confidence interval (CI) are reported for all regression analyses. A two-sided *P* value < 0.05 is considered to be statistically significant for all statistical tests. *P* values reported are rounded to three decimal places. All statistical analyses will be performed using SPSS 22.0 (SPSS Inc., Chicago, IL, USA).

## Results

During the study period, 42,976 women met the inclusion criteria; of them, 21,664 (50.4%), the study group, underwent episiotomy in their index delivery and 21,312 (49.6%), the comparison group, did not have episiotomy in the index delivery. Clinical and demographic characteristics of women with and without episiotomy in their first delivery are presented in Table 1. Table 2 presents that women with episiotomy in their previous vaginal deliveries had statistically significant higher rates of subsequent episiotomy (17.5 vs. 3.1%; *P* < 0.001; OR 1.9), the first and second degree perineal tears (33.6 vs. 17.8%; *P* < 0.001), the third and fourth degree perineal tears (0.2 vs. 0.1%; *P* = 0.002), and vacuum extractions (2.3 vs. 0.9%; *P* < 0.001). CD rate was comparable between the groups.

**Table 1:** Clinical and demographic characteristics for women with and without episiotomy in their index delivery

Characteristics	S/P episiotomy (*n* = 21,711)	No episiotomy (*n* = 21,355)	*P* value
Mother age mean ± SD	25.78 ± 4.548	25.77 ± 4.627	0.802
Ethnicity			< 0.001
Jewish	53.6% (11,638)	51.1% (10,916)	
Muslim	46.4% (10,026)	48.9% (10,396)	
Pregnancy age (weeks) mean ± SD	39.23 ± 1.937	38.99 ± 2.026	< 0.001
Birth weight (g)			< 0.001
< 2500	6.6% (1,424)	7.7% (1,645)	
2500–4000	89.5% (19,442)	88.8% (18,957)	
> 4000	3.9% (846)	3.5% (753)	
Birth weight (g) mean ± SD	3189.09 ± 529.936	3161.81 ± 507.777	< 0.001
Infant gender			0.138
Male	49.3% (10,693)	48.5% (10,365)	
Female	50.7% (10,971)	51.5% (10,947)	
Smoking	0.2% (52)	0.6% (127)	< 0.001
Polyhydramnios	3.2% (700)	1.7 (370)	< 0.001

**Table 2:** Obstetric outcomes in women with and without episiotomy on their subsequent delivery

Characteristics	S/P episiotomy (*n* = 21,711)	No episiotomy (*n* = 21,355)	*P* value
Episiotomy	17.5% (3808)	3.1% (656)	< 0.001
Cesarean delivery	5.8% (1270)	5.5% (1169)	0.095
Vacuum extraction	2.3% (498)	0.9% (198)	< 0.001
Perineal tear grade 1/2	33.6% (7290)	17.8% (3791)	< 0.001
Perineal tear grade 3/4	0.2% (43)	0.1% (18)	0.002

*C/S* cesarean section

While adjusting for maternal age, ethnicity, birth weight, and vacuum birth, the previous episiotomy was found to be an independent risk factor for repeated episiotomy in the subsequent delivery (adjusted OR 6.7; 95% CI 6.2–7.3, *P* < 0.001). The results remained significant (Table 3) for term (adjusted OR 6.8; 95% CI 6.2–7.4, *P* < 0.001) as well as preterm deliveries (adjusted OR 4.5; 95% CI 3.3–6.3, *P* < 0.001). While controlling for the pregnancy week: before and after 37 weeks, significantly higher rate of episiotomies was performed with infant weight above 4000 g; large for gestational age (LGA), compared with infant weight under 2500 g; small for gestational age (SGA); and higher rate of episiotomies with infants weight appropriate for gestational age (AGA) compared with SGA.

**Table 3:** Multivariate regression for episiotomy in consecutive pregnancy stratified by pregnancy week, before and after—37 weeks

Characteristics	OR	*P* value	95% CI
Before week 37			
S/P episiotomy	4.548	< 0.001	3.289–6.290
GDM	1.664	0.117	0.795–3.482
Infant weight			
<2500 vs. 2500–3999	0.93	0.615	0.703–1.232
PROM	0.707	0.113	0.450–1.111
Polyhydramnios	0.898	0.771	0.536–1.851
After week 37			
S/P episiotomy	6.8	< 0.001	6.224–7.430
GDM	1.302	0.008	1.070-1.584
Infant weight			
<2500 vs. 2500–3999	1.439	0.001	1.170–1.770
<2500 vs. > 4000	1.562	0.001	1.208–2.021
PROM	0.778	< 0.001	0.667–0.895
Polyhydramnios	1.599	< 0.001	1.347–1.897

## Discussion

Episiotomy is done in an effort to prevent soft-tissue tearing during labor which may involve the anal sphincter and rectum [12]. While the impact of episiotomy on the index delivery was investigated [2, 8], to the best of our knowledge, our study is the largest to investigate whether episiotomy in the index delivery influence the delivery and labor outcomes on subsequent delivery. Antonakou et al. found that women who had an episiotomy at first vaginal birth had an almost fourfold increased risk of repeat obstetric anal sphincter injury in a second vaginal birth [13].

The major finding of our study is that an association exists between episiotomy in the first vaginal delivery and higher rates of episiotomy and perineal tears in subsequent delivery. This association remained significant in two multivariate regression models. Moreover, episiotomy in the index labor was proven as a major risk factor for subsequent episiotomy in term as well as in preterm deliveries.

The explanations to the higher rate of episiotomy may rely in that (1) narrow woman anatomy causes the midwifes to have a tendency to perform a recurrent episiotomy. (2) Women who had episiotomy done before have a weaker scar tissue in this area which makes a preventive episiotomy more likely to be done to prevent perineal tears. (3) Women who give birth to higher birth weight infants, a known factor to make preventive episiotomy [14], tend to have heavier babies in the following pregnancies. (4) The pathophysiology of scar tissue created at the episiotomy site. Acute wounding alters the skin’s fibrotic structure, thereby producing scar tissue with significant functional impairments [15]. Scars showed significantly reduced failure properties (load, displacement, and energy), thus indicating their compromised bursting strength, extensibility, and toughness, with regard to uninjured skin [16, 17].

Our study major strength is its large population-based cohort that was retrospectively analyzed and the tertiary medical center being the one existing in the area. The weakness of the study may result from the retrospective design as well as from the inclusion criteria of our cohort; basically the inclusion of instrumental deliveries, in which episiotomy procedures are done routinely.

In conclusion, a significant association was found between episiotomy and perineal damage in the subsequent delivery. Women with a previous episiotomy were more likely to have an episiotomy and perineal tears on their subsequent delivery than women without the previous episiotomy. The results remained significant for term as well as preterm deliveries. Further studies should focus on different modalities to protect the perineum in this high-risk group of women.
